# Rapid detection method of bacterial pathogens in surface waters and a new risk indicator for water pathogenic pollution

**DOI:** 10.1038/s41598-023-49774-y

**Published:** 2024-01-18

**Authors:** Min Gao, Feiyang Tan, Yuan Shen, Yao Peng

**Affiliations:** College of Environmental and Chemical Engineering, Xi’an Key Laboratory of Textile Chemical Engineering Auxiliaries, Xi’an Polytechnic University, Xi’an, 710000 People’s Republic of China

**Keywords:** Environmental sciences, Hydrology

## Abstract

In this study, a accurate, rapid quantitative PCR method for the simultaneous detection of 4 kinds of pathogenic bacteria in water was established, and the distribution of pathogenic bacteria in surface waters with different levels of pollution (Yulin region, China) was detected. The results showed that the detection accuracy was 94%; the detection limit was 2.7 in bacterial cells. *Salmonella enterica subsp. enterica serovar typhimurium* and *Salmonella dysenteria* were always present in water when the universal primer for pathogenic bacteria abundance detection was greater than 10^4^ copies 100 mL^−1^. When the detection value is lower than 10^4^ copy 100 mL^−1^, the bacteria in the water are rarely pathogenic bacteria, so the detection value of 10^4^ copy 100 mL^−1^ can be used as a new indicator of waterborne pathogen pollution.

## Introduction

With the rapid development of urbanization, water pollution has become increasingly serious, leading to a rise in many diseases and the death rate of human beings, and these problems are particularly prominent in developing countries. In particular, some waterborne diseases, such as dysentery and enteritis, are caused by pathogenic bacteria^1^. Therefore, routine surveillance of pathogens in water is essential for public health. However, the current detection methods are slow and lack accuracy, which makes it difficult to prevent and control water-borne diseases.

During the past 40 years, three kinds of intestinal infectious diseases have occurred in China^2^. The first category is infectious diseases caused by bacteria, cholera, typhoid fever and bacterial dysentery. The second category is infectious diseases caused by pathogens and viruses, amoebic dysentery and hepatitis E (HEV). The third is infectious diseases caused by causes other than cholera, dysentery, typhoid and paratyphoid fever^3,4^. The annual average incidence of these diseases was 97.33 per 100,000 people^2^. In China, intestinal infectious diseases are mainly caused by *Escherichia coli (E. coli)*, *Shigella dysenteriae (S. dysenteriae)*, *Vibrio cholerae (V. cholerae)* and *Salmonella enterica subsp. enterica serovar typhimurium (S. typhimurium)*, accounting for over 50% of officially reported intestinal infectious diseases such as hepatitis A and typhoid fever^5,6^. In recent years, other kinds of infectious diarrhoea diseases have tended to rise in China. So, the detection of pathogenic bacteria in water bodies was imminent, but the risk of pathogenic bacteria causing diseases was not related to the index of assessment of routine regular water quality^7^.

At present, the detection of pathogenic bacteria in water is mainly based on selective culture and standard biochemical methods. However, this method has many defects. Firstly, the absence of colonies may occur during selective culture, but it does not mean that there are no viable cells, and there may be cells that have into the viable but non-cultureable (VBNC) state. Secondary, the species of the cells detected cannot be precisely located, and additional steps are required. Thirdly, very time-consuming^8–10^. Compared with traditional culture and biochemical methods, qPCR has the advantages of fast detection speed and low detection limit^11^.As a result, an increasing number of researchers have begun to solve this problem by molecular biological methods to shorten the time of monitoring and reporting^12–14^. One of these molecular biological methods is quantitative PCR, and this technique, together with probes and primers has been used widely for the monitoring of different faecal bioindicators and waterborne pathogenic bacteria^15–17^. At the same time, more studies have found correlation between bacterial culture and qPCR results on contaminated agar-like agar plates, which means that qPCR can be used as a superior alternative to culture in most detection scenarios^18–20^.

In this study, universal primers for 4 kinds of typical enteric pathogens including *E. coli*, *S. dysenteriae*, *V. cholerae* and *S. typhrmurium* were designed and the *E. coli* 16S rRNA gene was used as the target sequence. Specific primers for each of these four pathogenic bacteria have also been designed. The distribution of 4 kinds of pathogenic bacteria in the different surface waters located in Yulin city, northern Shaanxi Province, China, was monitored using qPCR with universal primers and specific primers. The sensitivity, accuracy and availability of this method in the range of the concentration of the whole environment were explored. The research results provided a feasible method to comprehensively evaluate the risk of surface water pathogenic pollution, which has important practical significance.

## Materials and methods

### Design and specificity of the primers

#### Primer design

Universal primers for *E. coli*, *S. dysenteriae*, *V. cholerae* and *S. typhimurium* were designed, and the *E. coli* 16S rRNA gene was used as the target sequence. Based on the high conservation of 16S rRNA genes of the above four pathogens, universal primers were designed. The sequences of the universal primers were as follows: 5′-aaggcgacgatccctagctggtctgagaggatga/c-3′ (246–280 bp, *E. coli*. 16S rRNA); 5'-gcttgccagtatcagatg cagttcccaggttgagc-3′ (521–556 bp, *E. coli*. 16S rRNA). The sequences of the specific primers for each of the 4 pathogenic bacteria are presented in Table 1. The synthesis of primers was completed by the Shanghai Bioscience & Technology Company, China.

**Table 1:** Sequences of the primers used in this study.

Target species	Primer sequence	References
*S. dysenteriae*	ipaH1 5′-GTTCCTTGACCGCCTTTCCGATAC-3′	^21^
	ipaH3 5′-CATTTCCTTCACGGCAGTGGA-3′	
*S. typhrmurium*	ST3 5′-AGATGGTACTGGCGTTGCTC-3′	^22^
	ST4 5′-TGGAGACTTCGGTCGCGTAG-3′	
*E. coli*	Es1 5′-TGTTCAGTGGCAAGAGTT-3′	^23^
	Es2 5′-TAATCGATATACCCGCTC-3′	
*V. cholerae*	ctxA1 5′-CTCAGACGGGATTTGTTAGGCACG-3′	^24^
	ctxA2 5′-TCTATCTCTGTAGCCCCTATTACG-3′	

#### Test of the primer specificity

The specificity test of the universal primers and the specific primers were completed by PCR and sequencing of the PCR products. DNA extracted from 14 reference species (Table 2) was used as the PCR template. The PCR amplification reaction system was composed of Taq DNA polymerase 1.0 U, dNTP 0.2 mmol L^−1^, 1 × PCR Buffer, 0.1 mmol L^−1^ upstream and downstream primer, 2.0 mmol L^−1^ MgCl_2_ and DNA template 2 μL, respectively, totaling 25 μL. PCR amplification conditions were as follows: denaturation at 94 ℃ for 5 min, 94 ℃ for 30 s, 55 ℃ for 30 s, 72 ℃ for 30 s, 35 cycles, and extension at 72 ℃ for 5 min. The DNA template was replaced with sterile ddH_2_O as the control group. PCR products were analysed by 2% agar sugar gelatine electrophoresis, which included 0.5 mg mL^−1^ bromize pyrimidine. A gelatine imaging system 1000 (Bio-Rad, USA) was used for imaging. The final PCR product was recovered and purified with a DNA Recovery Kit (Shanghai Bioengineering Company, China). Finally, the DNA fragment was sequenced (Shanghai Bioengineering Company, China), and then the homology of the sequence was analysed by DNA Star Software (Perkin Elmer, Norwalk, Connecticut, USA).

**Table 2:** Species and the sources.

No.	Species	Source	No.	Species	Source
EF424586	*Staphylococcus aureus*	SXW	NC_009089	*Clostridium Difficile*	SXW
EF420247	*Bacillus subtilis*	SXW	EF421208	*Bifidobacterium longum*	SXW
EF378646	*Pseudomonas aeruginosa*	SXW	DQ171719	*Lactococcus lactis*	SXW
EF422070	*Bacillus cereus*	SXW	DQ362495	*Shigella dysenteriae*	ATCC
EF413067	*Bacillus thuringiensis*	SXW	DS179652	*Vibrio cholerae*	ATCC
EF394153	*Bradyrhizobium japonicum*	SXW	D12814	*Salmonella enterica subsp. enterica serovar typhimurium*	ATCC
BD267944	*Lactobacillu bulgaricus*	SXW	EF418614	*Escherichia coli*	ATCC

*SXW* Institute of Microorganism in Shaanxi Province, *ATCC* American Type Culture Collection.

### qPCR detection method

#### Bacterial cultivation

The reference and standard species used in this study (Table 2) were obtained from the American Type Culture Collection (ATCC) and Institute of Microorganism in Shaanxi Province (SXW). LB medium include peptone 10 g L^−1^ (Thermo Fisher Scientific Company, China), NaCl 5 g L^−1^, yeast extract 5 g L^−1^ (Shanghai Yuanye Biotechnology Company, China) contained in each litre of water was chosen as the culture medium. The bacteria were incubated at 37 ℃ for 24 h, and then centrifuged at 10,744 ×*g* (Hunan Xiangyi Laboratory Instrument Development Company, xiangyi H1850R, China). After that, cells were collected as described by the American Public Health Association^25^.

#### qPCR standard curve

The four kinds of pathogenic bacteria cultures with clear cell density were diluted 10 times with sterile distilled water, and then the diluted bacterial cultures were centrifuged at 10,744 ×*g* for 10 min. After bacterial cells were reclaimed and washed with sterile distilled water 3 times, total DNA was extracted by the phenol–chloroform method. The total DNA was taken as the template for the preparation of the qPCR standard curve. Three concentrated groups were analysed, and the bacterial cell density of each diluted bacterial culture was determined by counting the average of 3 qPCR detection results^26^.

### qPCR analysis system

The qPCR system was formed after adding the fluorescence reagent SYBR Green I (Tiangen Biological and Chemical Company, China) based on of the above PCR system. Then the PCR process was detected in real time by detecting the fluorescence signal. The qPCR reaction system was composed of 2 μL DNA template, 1 × real MastrMix/1 × SYBR solution, and 0.1 mmol L^−1^ upstream and downstream primers, with a volume of 25 μL. The qPCR mixing reaction liquid was placed into an 8-position tube (MJ Research TLS-0251), sealed with a super clean lid, and then placed into a qPCR instrument (American Bio-Rad MJ). The amplification conditions of qPCR were predenaturation at 94 ℃ for 30 s, 55 ℃ for 30 s, 72 ℃ for 30 s, and 85 ℃ for 2 s, for 35 cycles. After manually adjusting the threshold fluorescence value to 8 units, the instrument automatically measured the cycle threshold (C_T_)^21^.

### Detection of pathogenic bacteria in surface waters

#### Sampling point and sampling time

Clean waters (source water), slightly polluted waters (once polluted river was treated; natural lake) and seriously polluted waters (water body that accepts contaminant; effluent from wastewater treatment plant) were chosen as research objects. The water quality of the surface waters mentioned above id presented in Table 3.

**Table 3:** Water quality of the different surface waters located in Yulin city.

Sampling site	WWTP effluent A	River B	River C	Lake D	River E
Turbidity (NTU)	2.4–13.1	11.1–747.0	6.1–67.0	8.1–75.0	1.7–16.3
pH	7.1–8.4	7.4–8.3	7.3–8.8	6.7–9.8	8.1–8.7
DO (mg L^−1^)	2.2–7.6	0.6–4.3	7.8–13.5	3.8–11.2	8.1–12.4
Chloride (mg L^−1^)	62.7–85.8	9.6–43.6	2.5–5	8.5–21.4	0.2–0.7
TP (mg L^−1^)	0.4–2.9	0.07–1.4	0.04–0.5	0.1–2.5	0.02–0.3
TN (mg L^−1^)	1.3–19.4	2.0–13.4	0.4–1.0	0.1–2.2	0.2–0.7
COD (mg L^−1^)	30.4–65.7	13.3–65.7	7.3–22.9	27.1–68.9	2.3–8.7
Uses of the water	Secondary effluent	A river received sewage	A river which was treated in recent years	Natural desert lake	Drinking water source
Remark	Without disinfection	Seriously polluted	Slightly polluted	Slightly polluted	Clean

Except for some bad weather, samples were collected in the following five places: wastewater treatment plant A, river B, river C, river E and lake D. Samples were collected 4 times each month for one year (from March 2015 to March 2016). Water samples (500 mL) were collected at half the depth of each water body in the morning (from 8:00 to 12:00) and other auxiliary materials including pH, temperature, turbidity, tourist density and something about boats (type, quantity, et al.) were collected as described by the American Public Health Association^25^.

#### Total DNA extraction

A 100 mL surface water sample was centrifuged at 10,744 ×*g* for 10 min, and then the supernatants was removed. Next, 567 μL of broken buffer, which was composed with of 40 mmol L^−1^ Tris–HCl (Shanghai Yuanye Biotechnology Company, China), pH 8.0, 20 mmol L^−1^ CH_3_COONa, 1 mmol L^−1^ EDTA (Shandong Yinuo Chelating Chemical Company, China), and 1% SDS (Hangzhou Lianke Meixun Biomedical Technology Company, China), was added to the centrifuged sediment to resuspend the sediment. Then, 66 μL of 5 mol L^−1^ NaCl was added, adequately mixed at 65 ℃ for 20 min, and centrifuged (10,744 ×*g*) for 10 min. The supernatant after centrifugation was transferred to a new tube, and an equal volume mixture was added for extraction, which consisted of isochoric phenol (Shaanxi Jintai Chlor-alkali Chemical Company, China), chloroform (Shaanxi Sinopharm Medical Diagnostic Reagent Company, China), and isoamyl alcohol (Nanjing Oriental Pearl Industry and Trade Company, China)at a ratio of 25:24:1. The mixture in the previous step was centrifuged (10,744 ×*g*) for 5 min, the supernatants were taken and 0.6 times the volume of isoamyl alcohol was added to obtain DNA sediment. The obtained DNA precipitate was washed with 1 mL of 70% ethanol 3 times, and the ethanol was removed after centrifugation. Finally, the DNA was shunted with ddH_2_O and stored for PCR amplification^27^.

#### qPCR analysis of the surface water samples

At least three replicate pathogenic bacterial DNA extracts in environmental water should be ready, and the cell copies determined by qPCR in diluted water samples multiplied by the dilution coefficient were taken as the cell density, with a range of 10^1^–10^7^. The scatter plot and regression analysis of log_10_ cell copies of bacteria on qPCR-measured cycle threshold (C_T_) values for serially diluted DNA extracts of bacteria were used to evaluate the sensitivity and precision of the qPCR method^28^. The cell density of pathogenic bacteria in environmental water samples was calculated according to the known standard curve established by cell density and qPCR. The calculated value is a relative quantity.

## Results and discussion

### Design of the PCR universal primers for typical bacterial pathogens in water

**Figure 1:**
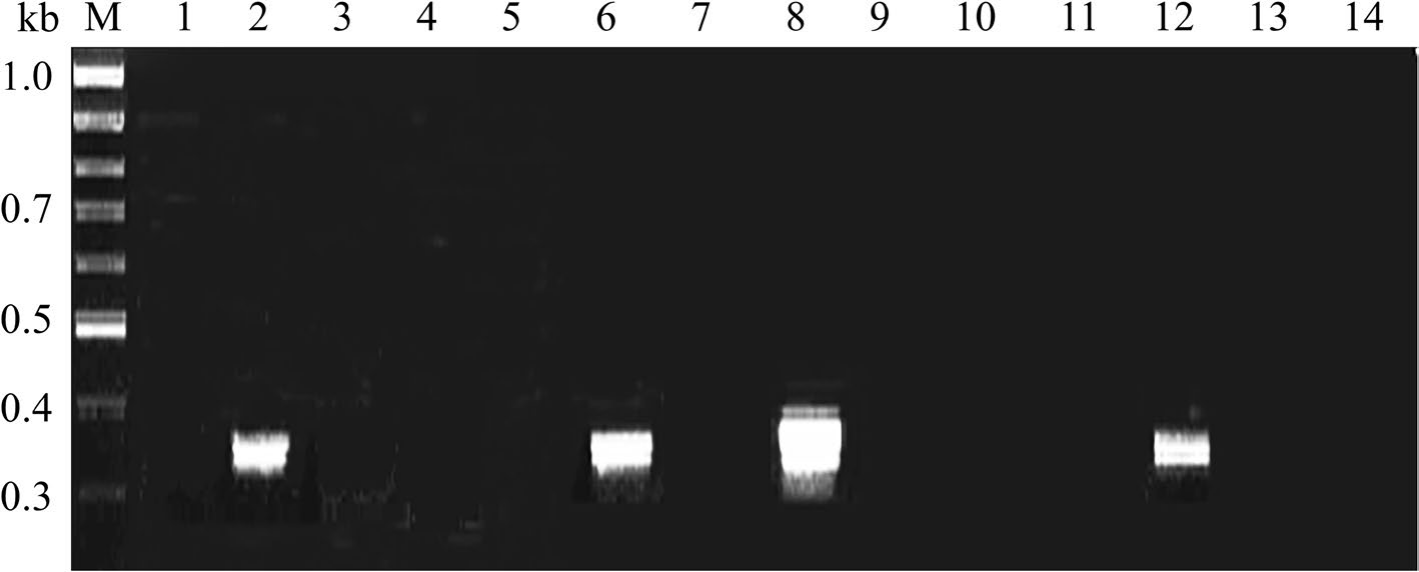
PCR amplification results of the 4 target strains and 10 reference strains. M: DNA Marker; 2: *S. dysenteriae*; 6: *V. cholerae*; 8: *S. typhimurium*; 12: *E. coli*; 1, 3–5, 7, 8–11, 13–14: reference strains.

The 16S rRNA gene sequences of *E. coli*, *S. dysenteriae*, *V. cholerae* and *S. typhimurium* were searched in GenBank. By analysis of homology using DNA-STAR software (version 3.2), the universal primers were designed as 5′-aaggcgac gatccctagctggtctgagaggatga/c-3′ (nt 246–280 dp, *E. coli* 16S rRNA numbering), and 5′-gcttgccagtatcagatgcagttcccaggttgagc-3′ (nt 521–556 dp, *E. coli* 16S rRNA numbering). The universal primer and the specific primers for the 4 kinds of pathogenic bacteria mentioned above were tested by PCR. As shown in Fig. 1 (original blots/gels are presented in Supplementary Fig. S1), a limpid specific strap could be seen at 320 bp for the 4 strains on the electropherograms, while no amplification products were identified for the 10 reference strains. The analysis results verified that the universal primers showed specificity only for their corresponding target genes. After purification, the amplified products of the nucleotide sequence were detected. Compared with the 4 kinds of pathogens in the GenBank of the 16S rRNA gene sequence, the similarity of sequencing results was more than 99%. This proved that the universal primer designed in this study was suitable for the specific detection of most general species of the four target strains.

**Figure 2:**
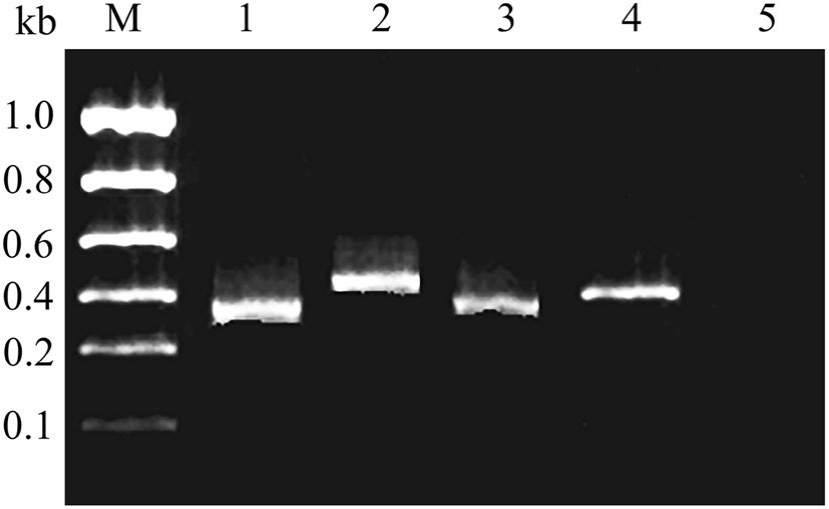
PCR amplification results of the 4 target strains with specific primers. M: DNA Marker, 1: *S. dysenteriae,* 2: *V. cholerae*, 3: *S. typhimurium*, 4: *E. coli*, 5: Negative control.

The nucleotide sequence results for the amplified products also showed that the 4 pairs of specific primers used in the research had very high specificity to the target strains (Fig. 2, original blots/gels are presented in Supplementary Fig. S2).

### Establishment of the qPCR method for the detection of bacterial pathogens in water

**Figure 3:**
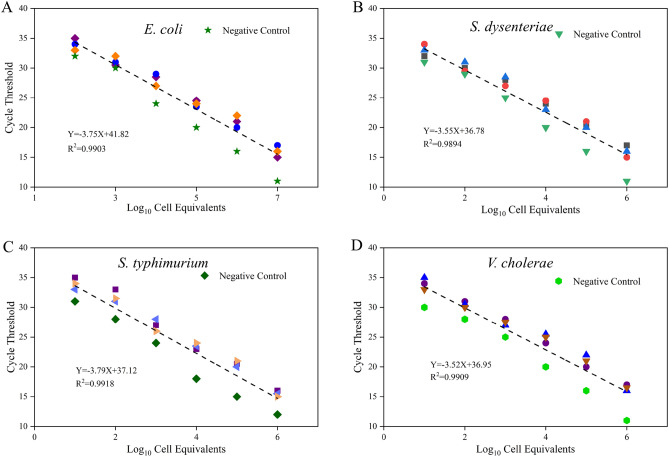
Scatter plot and regression analysis results of log_10_ cell copies of *E. coli* (**A**)*, S. dysenteriae* (**B**)*, S. typhimurium* (**C**) *and V. cholerae* (**D**) on qPCR-measured cycle threshold (C_T_) values for serially diluted DNA extracts from them. Negative control: contains everything except pathogen DNA target (including primers).

The relationship of the bacterial pathogens in water between the qPCR-measured cycle threshold (C_T_) values and log_10_ cell copies from three concentrate parallel samples as shown in Fig. 3. By obtaining the C_T_ value of the water sample, the original bacterial pathogens cell density of the water sample can be calculated according to the standard curve of qPCR. The slope of the equation could be used to examine the efficiency of PCR, the slope of *E. coil, S. dysenteriae, V. cholerae, and S. typhimurium* were − 3.75, − 3.55, − 3.79 and − 3.52, respectively. A perfect qPCR standard curve was based on the PCR efficiency reaching 90–100% (100% PCR efficiency means that the quantity of DNA template will be doubled after each cycle). Only when the linear regression analyses of the standard curve had a high correlation coefficient (R^2^ ≥ 0.99), would the process and data of the qPCR experiment be believable^26,29,30^.

The original DNA template copies of *E. coli* examined in this experiment ranged from 6.8 × 10^1^ to 6.8 × 10^5^ cfu mL^−1^, and the log_10_ cell copies of *E. coli* DNA template were positively proportional to its corresponding C_T_ value (Fig. 3A). The regression coefficient was 0.9903, and it showed a strong linear relation, the slope was − 3.75 and the PCR efficiency was 99%. The standard deviation of the C_T_ value between replicated DNA extracts of every diluted grade was less than 0.3. Cell copies of *S. dysenteriae*, *V. cholerae*, and *S. typhimurium* (whose cells densities were known) were examined by qPCR with universal primers. The scatter plot and regression analysis results indicated that the slope was not changed or interrupted (Fig. 3B–D) and the reliability of the examination result by the qPCR method was 94%.

**Figure 4:**
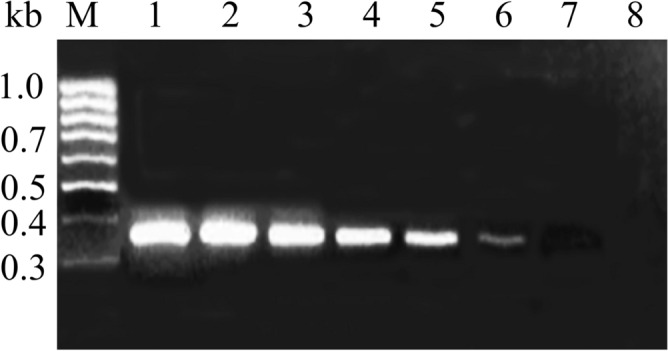
Sensibility of the qPCR detection by using the universal primer: qPCR amplification results of the *E. coli* cultures serially tenfold diluted with sterile distilled water. M: Marker, 1: 2.7 × 10^5^ cfu 100 mL^−1^; 2: 2.7 × 10^4^ cfu 100 mL^−1^; 3: 2.7 × 10^3^ cfu 100 mL^−1^; 4: 2.7 × 10^2^ cfu 100 mL^−1^; 5: 2.7 × 10^1^ cfu 100 mL^−1^; 6: 2.7 × 10^0^ cfu 100 mL^−1^; 7: 2.7 × 10^–1^ cfu 100 mL^−1^; 8: Negative control.

Strain *E. coli* was taken as a representative strain, and the sensitivity of the qPCR method with universal primers was examined. The results showed that the minimum detection limit of the qPCR method (including the process of bacterial cell reclamation, DNA extraction method and qPCR method) was 2.7 bacterial DNA extracts (Fig. 4, original blots/gels are presented in Supplementary Fig. S3). It shows that the detection limit of the universal primers is low, which can meet the detection needs under low concentrations of pathogenic bacteria.

### Distribution of pathogenic bacteria in the surface waters

Universal primers for bacterial pathogens and specific primers for 4 kinds of typical pathogenic bacteria were used to detect pathogenic bacteria by qPCR in five surface water samples, clean water, lightly polluted water and seriously polluted water. Detection was continued for one year, and 4 samples, were collected each month. The detection results of pathogenic flora, *S. dysenteriae*, *V. cholerae*, *S. typhimurium* and *E. coli* in different surface waters are shown in Table 4. The number of samples for each water body was 48 (n = 48).

**Table 4:** Cell densities of pathogenic bacteria detected by using universal primer and specific primer, respectively in surface waters (copies 100 mL^−1^).

Sampling site	WWTP effluent A	River B	River C	Lake D	River E
No. of samples (n = 240)	(n = 48)	(n = 48)	(n = 48)	(n = 48)	(n = 48)
*E. coli* (specific primer)					
Max	1.6 × 10^5^	2.5 × 10^5^	2.2 × 10^1^	2.6 × 10^2^	1.6 × 10^1^
Min	1.8 × 10^4^	2.0 × 10^3^	0	1.0 × 10^1^	0
Mean	5.0 × 10^4^	9.1 × 10^4^	7.2	6.7 × 10^1^	2.7
*S. typhrmurium* (specific primer)					
Max	1.3 × 10^2^	3.0 × 10^2^	2.1 × 10^1^	3.0 × 10^1^	1
Min	2.1 × 10^1^	2.5 × 10^1^	1	1.0	0
Mean	6.3 × 10^1^	9.2 × 10^1^	5.2	6.0	0.7
*S. dysenteriae* (specific primer)					
Max	2.5 × 10^2^	3.3 × 10^2^	2.1 × 10^1^	8.9 × 10^1^	2.7 × 10^1^
Min	3.0	2.0	0	0	0
mean	3.3 × 10^1^	4.6 × 10^1^	2.5	1.5 × 10^1^	4.0
*V. cholera* (specific primer)					
Max	0	0	0	0	0
Min	0	0	0	0	0
Mean	0	0	0	0	0
Pathogenic flora (universal primer)					
Max	3.2 × 10^5^	1.1 × 10^6^	7.4 × 10^3^	4.4 × 10^3^	1.9 × 10^3^
Min	1.9 × 10^4^	2.1 × 10^4^	5.2 × 10^2^	1.8 × 10^3^	1.5 × 10^2^
Mean	1.1 × 10^5^	3.0 × 10^5^	3.3 × 10^3^	3.1 × 10^3^	8.5 × 10^2^

*V. cholerae* was not found in any of the detected waters. *V. cholerae* was not present in the surface waters of the Yulin area under normal conditions. In clean water (E), the average values of enteric pathogenic flora were 850 copies 100 mL^−1^, and the average values of *E. coli*, *S. dysenteriae* and *S. typhimurium* were 2.7 copies 100 mL^−1^, 4.0 copies 100 mL^−1^ and 0.7 copies 100 mL^−1^, respectively. Water body E was the source water of Yulin city, so it was well protected, and was not polluted by pathogens.

In the lightly polluted water (C, D), the average values of pathogenic flora were (3.1–3.3) × 10^3^ copies 100 mL^−1^, and the average values of *E. coli*, *S. dysenteriae* and *S. typhimurium* were 7.2–67 copies 100 mL^−1^, 2.5–15 copies 100 mL^−1^ and 5–6 copies 100 mL^−1^, respectively. The total number of 4 kinds of typical pathogenic bacteria detected by specific primers was far less than the number of pathogenic flora detected by universal primers. In the seriously polluted water (A, B), the average values of pathogenic flora were (1.1–3.0) × 10^5^ copies 100 mL^−1^, concentrations of *E. coli* were very high, and the average value was 5–9.1 × 10^4^ copies 100 mL^−1^. The average values of *S. dysenteriae* and *S. typhimurium* were 33–46 copies 100 mL^−1^ and copies 100 mL^−1^, respectively. The total cell intensity of 4 kinds of pathogenic bacteria detected by specific primers is close to the pathogenic flora detected by universal primers. It shows that the universal primers designed in this study are similar in accuracy to the specific primers of the four pathogenic bacteria.

### Positive rate of pathogenic bacteria in surface waters

Among the 240 samples from the five surface waters, the detection frequency of *V. cholerae* was 0, which is the universal detection frequency of pathogenic flora (100%) (Table 5). In the different surface waters, the detection frequencies of *E. coli*, *S. dysenteriae* and *S. typhimurium* were different. The positive rate of *E. coli* and *S. typhimurium* was 100% in the seriously polluted waters (A, B) and lightly contaminated waters (C, D). However, in the clean water (E), the positive rates of *E. coli* and *S. typhimurium* were 72.2% and 27.3%, respectively.

**Table 5:** Positive rates of bacterial pathogens in surface waters.

Target organisms	Positive rate of pathogenic bacteria (%), (n = 55)				
	WWTP effluent A (n = 48)	River B (n = 48)	River C (n = 48)	Lake D (n = 48)	River E (n = 48)
*E. coli* (specific primer)	100	100	100	100	72.7
*S. typhrmurium* (specific primer)	100	100	100	100	27.3
*S. dysenteriae* (specific primer)	91.9	100	18.2	72.7	18.2
*V. cholera* (specific primer)	0	0	0	0	0
Pathogenic flora (universal primer)	100	100	100	100	100

In the seriously polluted water, the detection frequency of *S. dysenteriae* was up to 90%, but in the lightly contaminated water and clean water, the detection frequency of *S. dysenteriae* was only 18.2%. When the waters were slightly polluted, *E. coli* could be detected out continuously, when the waters were polluted seriously, *S. typhimurium* and *S. dysenteriae* could be detected continuously, indicating that the pollution degree of pathogens was closely related to the degree of water pollution^31–33^.

### Contamination risk analysis of pathogenic bacteria in surface waters

**Figure 5:**
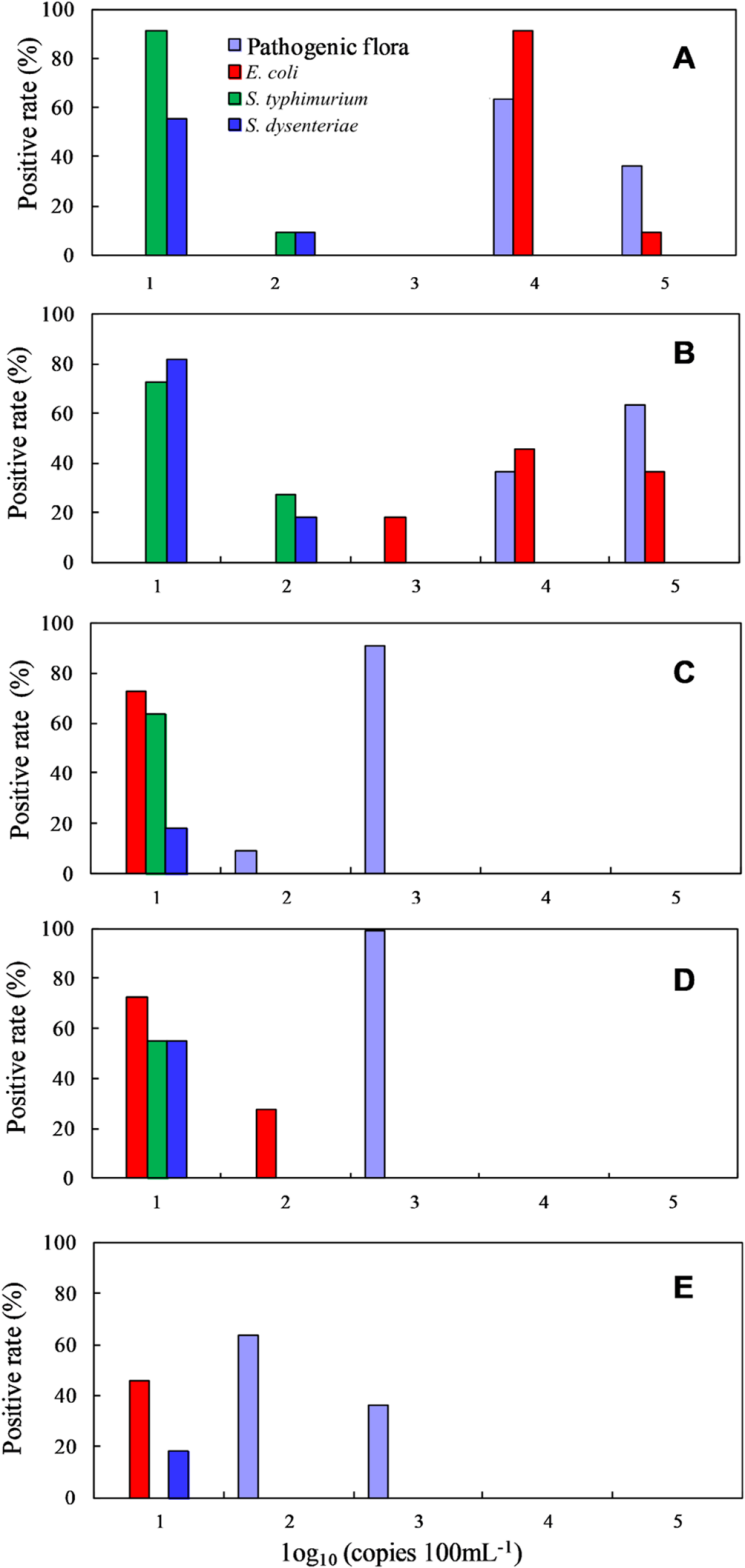
Distribution of the positive rate of pathogenic bacteria in the different surface waters. (**A**) Secondary effluent of the wastewater treatment plant (without disinfection), (B) a river that received sewage (seriously polluted), (**C**) a river that was treated in recent years (slightly polluted), (**D**) natural desert lake (slightly polluted), (**E**) drinking water source (clean).

Figure 5 shows the distribution of the positive rate of pathogenic flora detected by universal primers and 4 kinds of pathogenic bacteria detected by specific primers in different surface waters. In seriously polluted water (Fig. 5A,B), the cell density of pathogenic flora detected by universal primers was 10^4^–10^5^ copies 100 mL^−1^, and the positive rate at this concentration was over 50%. *S. typhimurium* and *S. dysenteriae* could also be detected continuously, and the cell densities were 10^1^–10^2^ copies 100 mL^−1^. For seriously polluted water, when the detection value of universal primers was greater than 10^4^ copies 100 mL^−1^, although the main pathogenic flora in water was *E. coli*, there must be the presence of *S. typhimurium* and *S. dysenteriae* in water, and the risk of pathogen contamination increased greatly.

In lightly polluted water (Fig. 5C,D) or clean water (Fig. 5E), the cell density of pathogenic flora detected by universal primers was 10^2^–10^3^ copies 100 mL^−1^, the cell density of all 4 kinds of typical pathogenic bacteria together was approximately 10 copies 100 mL^−1^, and these pathogenic bacteria could not be detected in succession. The positive rates of 4 kinds of typical pathogenic bacteria were far lower than the positive rates of pathogenic flora detected by universal primers, indicating that when the cell density of pathogenic flora was lower than 10^4^ copies 100 mL^−1^ in water, the pathogenic bacteria that were detected by universal primers were mainly bacteria other than *E. coli*, *S. typhimurium*, *S. dysenteriae* and *V. cholera*. The results showed that the generic primers in this study can be better used as detection tools than specific primers, and when the cell density detected by the universal primers was higher than 10^4^ copies of 100 mL^−1^, the risk of bacterial pathogen contamination in surface water was significantly increased, and 10^4^ copies of 100 mL^−1^ was recommended as a new indicator of water-induced contamination.

### Correlation analysis of the pathogenic bacteria detected by using universal primers and specific primers

**Figure 6:**
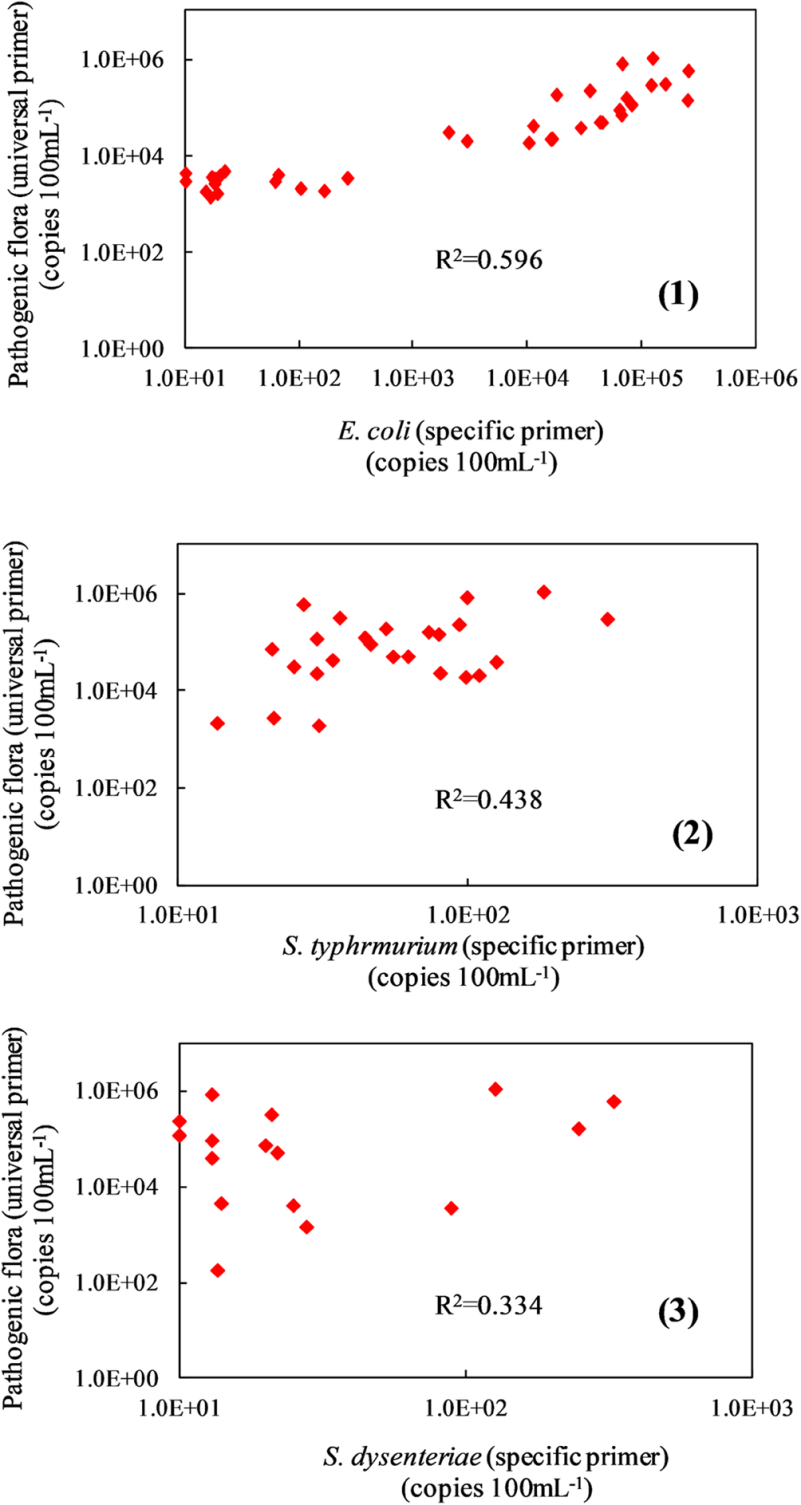
Scatter plot and regression analysis of pathogenic flora detected by using universal primers and specific primers.

The scatter plot and regression analysis of pathogenic flora detected by universal primers and 4 kinds of typical pathogenic bacteria detected by specific primers in the surface water are shown in Fig. 6^34.^

It was concluded that the coefficient between pathogenic flora and *E. coli*, *S. typhimurium* and *S. dysenteriae* were r = 0.77, r = 0.66 and r = 0.58, respectively. The high correlation indicates that the accuracy of detection between generic and specific primers in this study is similar. There was a strong correlation between pathogenic flora and *E. coli*, indicating that the main enteric pathogenic flora was *E. coli* in the surface water of Yulin city. There results are consistent with the pollution situation of water pathogens tested by traditional methods of coliform indicators.

The qPCR method based on universal primers of enteric pathogenic flora could detect not only *E. coli* in water, but also *S. dysenteriae*, *S. typhimurium*, and *V. cholera*. Compared with the traditional methods, the qPCR method could better reflect the true situation of water pathogen pollution. In addition, it has many advantages, such as high sensitivity and short test time (less than 5 h). In the routine detection of pathogens in surface water, although the qPCR method cannot completely replace the traditional method of detection of *E. coli* at present because the two methods do not fully correspond^35–37^, the detection results by the qPCR method in this study could be used as an important reference index for the risk assessment of pathogenic contamination in surface water.

## Conclusions and recommendations

In this study, a qPCR method with high sensitivity, short detection time and high accuracy was established, which could simultaneously detect four pathogenic bacteria (*E. coli, S. dysenteriae, V. cholerae* and *S. typhimurium*) in five typical water samples. Compared to traditional specific primers, the qPCR method could be finished within 5 h, the detection accuracy was 94%, and the detection limit was the amount of DNA extracted from 2.7 cells. The results showed that *V. cholera* did not exist in the Yulin region in general. The positive rate of *E. coli* was the highest in all kinds of waters, and it could be detected continuously when the water was slightly polluted. For seriously polluted water, *E. coli* was the main pathogenic bacteria. When the cell density of pathogenic flora detected by using universal primers exceeded 10^4^ copies 100 mL^−1^, *S. typhimurium* and *S. dysenteriae* could be detected continuously. When the cell density of pathogenic flora was less than 10^4^ copies 100 mL^−1^, the bacteria detected in the water were bacteria other than 4 kinds of typical pathogens, which meant that 10^4^ copies 100 mL^−1^ could be considered one of the important signs of water pathogenic pollution. Among the five typical water samples in this study, it has good universality. However, the universality of qPCR method in water samples with more serious pollution and more complex forms of pollution needs further study.

### Supplementary Information

Supplementary Figures.

## Data Availability

The data presented in this study are available in the article.
