# Supplementary material for: Rapid detection method of bacterial pathogens in surface waters and a new risk indicator for water pathogenic pollution
Source: Sci Rep. 2024 Jan 18;14:1614. doi: 10.1038/s41598-023-49774-y (PMC10796392; doi:10.1038/s41598-023-49774-y)
Supplement: Supplementary file 1 — Supplementary Figures. [file 41598_2023_49774_MOESM1_ESM.docx]

Rapid detection method of bacterial pathogens in surface waters and a new risk indicator for water pathogenic pollution

Min Gao*, Feiyang Tan, Yuan Shen and Yao Peng

College of Environmental and Chemical Engineering, Xi'an Key Laboratory of Textile Chemical Engineering Auxiliaries, Xi'an Polytechnic University, Xi'an, 710000, PR China.

*****Correspondence: author: Tel: +86-29-62779281, Fax: +86-29-62779281, E-mail: 20200404@xpu.edu.cn


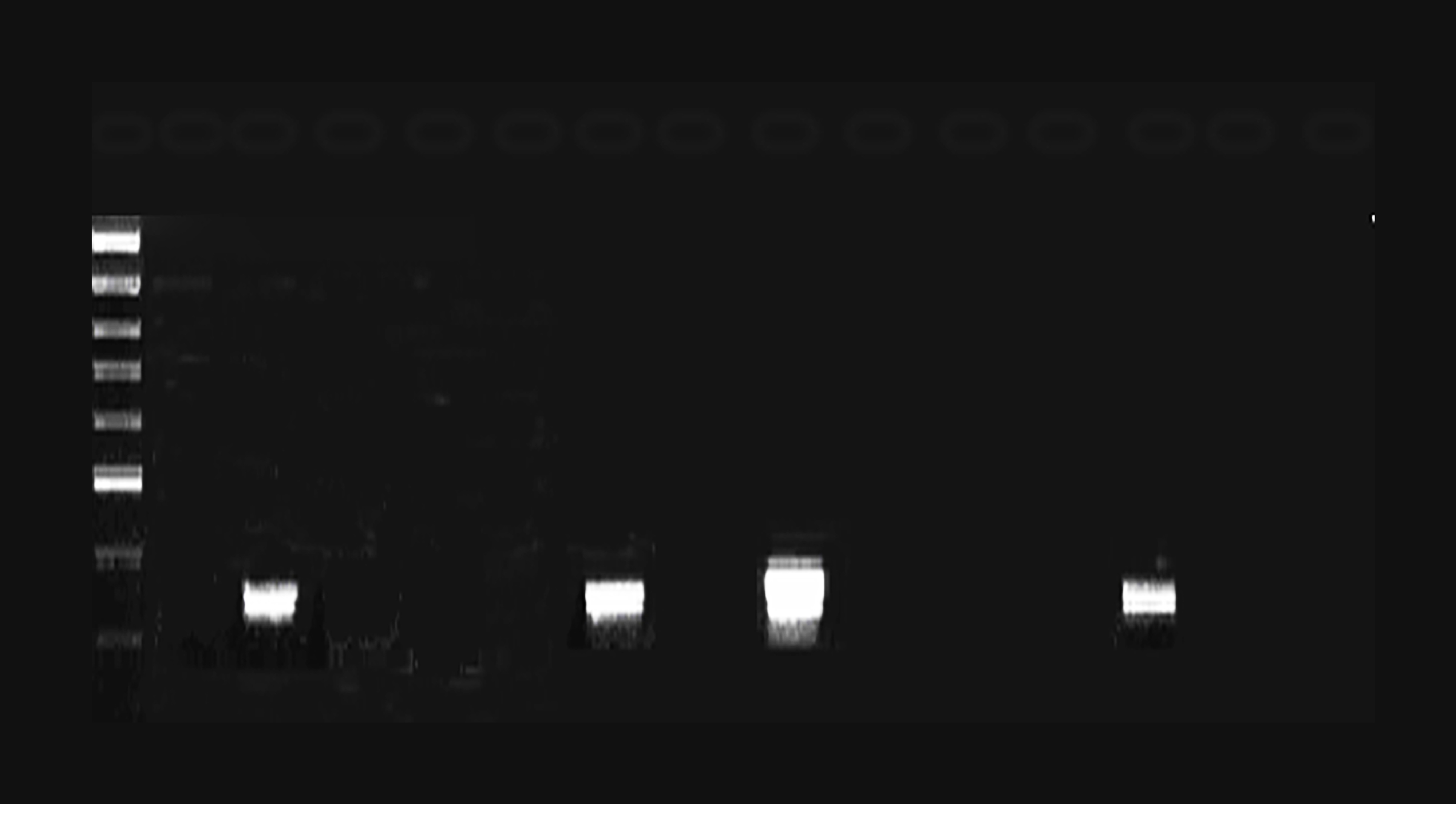


**Supplementary Figure S1.** Original blots/gels in **Fig. 1.** PCR amplification results of the 4 target strains and 10 reference strains.


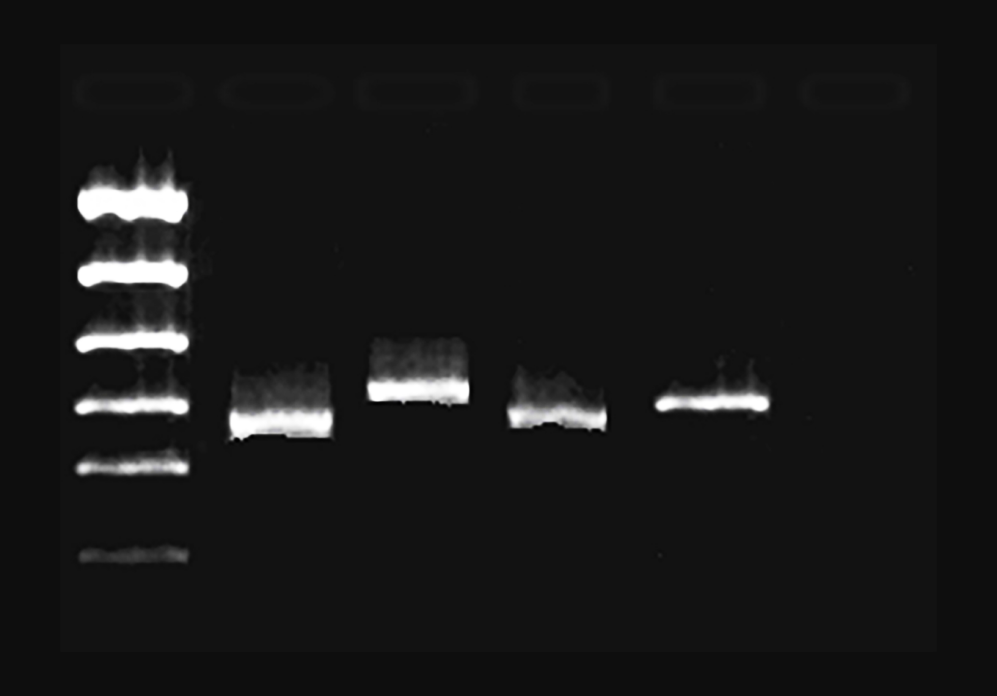


**Supplementary Figure S2.** Original blots/gels in **Fig. 2.** PCR amplification results of the 4 target strains with specific primers.


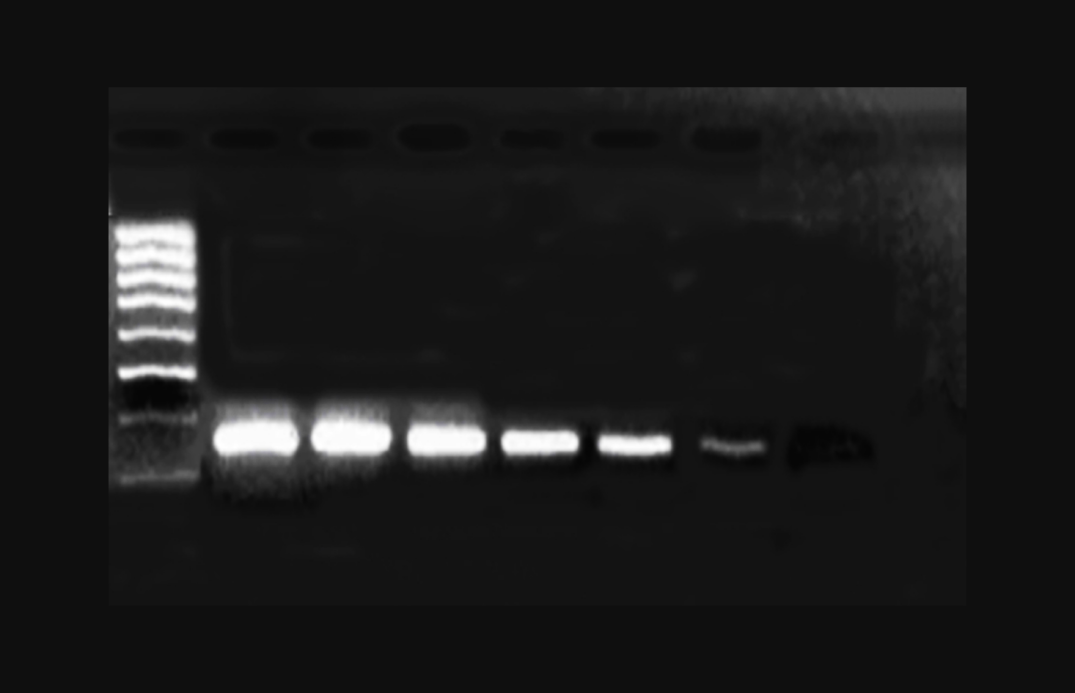


**Supplementary Figure S3.** original blots/gels in **Fig. 4.** Sensibility of the qPCR detection by using the universal primer: qPCR amplification results of the *E. coli* cultures serially tenfold diluted with sterile distilled water.
